# Supplementary material for: Identification of Freezing-Responsive microRNAs and Their Targets in Chinese Jujube by Small RNA and Degradome Sequencing
Source: Int J Mol Sci. 2026 Sep 4;27(17):7912. doi: 10.3390/ijms27177912 (PMC13566890; doi:10.3390/ijms27177912)
Supplement: Supplementary file 1 [file ijms-27-07912-s001.zip › Supplementary Material S2/novel_miR_179.pdf]

Provisional ID	:	novel_miR_179
Score total	:	2.5
Score for star read(s)	:	-1.3
Score for read counts	:	0
Score for mfe	:	2.2
Score for randfold	:	1.6
Score for cons. seed	:	
Total read count	:	114
Mature read count	:	114
Loop read count	:	0
Star read count	:	0

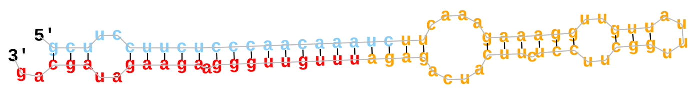

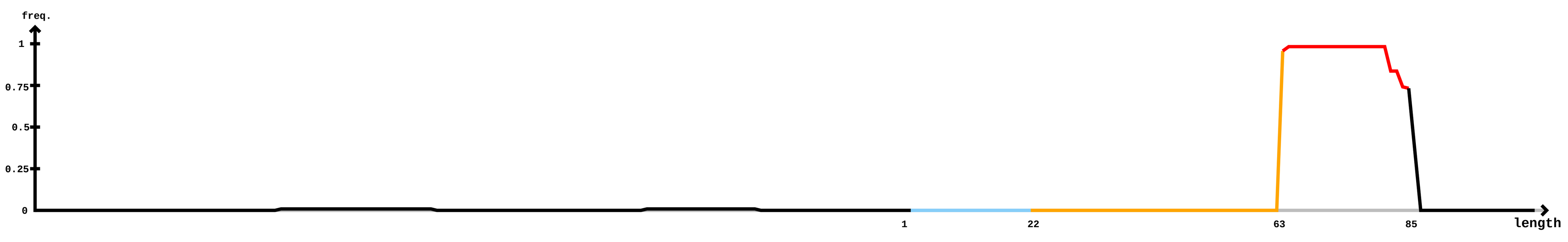


```
                                                                                                                                                                   Star                                                   Mature
5'- auccaacuuggauaauaauauacuuuggccagaagaguuuguauauauauauauauauauauauauauauaaauguauauauugagccaaaguuaguauuugaagagucucgaaagaaggaagaucaucuucucugccaugccugcuuccuucucccaacaaaucuucaaagaaagguuguuauuuggcuuccucuucaucagagauuuguugggaagaagauagcagagauugcagauaagagaacgac -3'   exp
    (((((...)))))...((((((((((((((((....(((((((((((((((((....))))))))))))))))).......))).))))))))..))))).((((((((((........).))))..)))))(((((.((..(((((..((((((((((((((((((....((((((..(((....)))..))).)))....))))))))))))).)))))..)))))..)).))))).............   reads   mm   sample
    .......................................uguauCuauauauauauauauauaua.........................................................................................................................................................................................   1       1    LCK-3
    ....................................................................................................ugaagagGcucgaaagaag...................................................................................................................................   1       1    LT-2
    ..............................................................................................................................................................................................................uuuguugggaagaagaua..........................   1       0    JCK-1
    ..............................................................................................................................................................................................................uuuguugggaagaagaua..........................   6       0    JT-3
    ..............................................................................................................................................................................................................uuuguugggaagaagaua..........................   1       0    JCK-2
    ..............................................................................................................................................................................................................uuuguugggaagaagaua..........................   3       0    LT-2
    ..............................................................................................................................................................................................................uuuguugggaagaagaua..........................   5       0    JT-2
    ..............................................................................................................................................................................................................uuuguugggaagaagaua..........................   1       0    JCK-3
    ..............................................................................................................................................................................................................uuuguugggaagaagauagc........................   2       0    JCK-3
    ..............................................................................................................................................................................................................uuuguugggaagaagauagc........................   1       0    LT-1
    ..............................................................................................................................................................................................................uuuguugggaagaagauagc........................   2       0    JCK-2
    ..............................................................................................................................................................................................................uuuguugggaagaagauagc........................   5       0    JCK-1
    ..............................................................................................................................................................................................................uuuguugggaagaagauagc........................   1       0    LT-3
    ..............................................................................................................................................................................................................uuuguugggaagaagauagca.......................   1       0    LT-3
    ..............................................................................................................................................................................................................uuuguugggaagCagauagcag......................   1       1    JCK-3
    ..............................................................................................................................................................................................................uuuguugggaagaagauagcag......................   20      0    JCK-3
    ..............................................................................................................................................................................................................uuuguugggaagaagauCgcag......................   1       1    JCK-1
    ..............................................................................................................................................................................................................uuuguugggaagaagauagcag......................   26      0    JCK-1
    ..............................................................................................................................................................................................................uuuguugggaagaagauagcag......................   34      0    JCK-2
    ...............................................................................................................................................................................................................uuguugggaagaagauagcaga.....................   1       0    JCK-3
    ...............................................................................................................................................................................................................uuguugggaagaagauagcaga.....................   1       0    JCK-1
    ...............................................................................................................................................................................................................uuguugggaagaagauagcaga.....................   1       0    JCK-2
```
